# Routine abdominal drainage after distal pancreatectomy: meta-analysis

**DOI:** 10.1093/bjs/znac042

**Published:** 2022-03-30

**Authors:** Eduard A van Bodegraven, Tess M E van Ramshorst, Alberto Balduzzi, Mohammed Abu Hilal, I Quintus Molenaar, Roberto Salvia, Casper van Eijck, Marc G Besselink

**Affiliations:** Department of Surgery, Amsterdam UMC, University of Amsterdam, Cancer Centre Amsterdam, Amsterdam, the Netherlands; Department of Surgery, Amsterdam UMC, University of Amsterdam, Cancer Centre Amsterdam, Amsterdam, the Netherlands; Department of Surgery, Poliambulanza Hospital Brescia, Brescia, Italy; Department of General and Pancreatic Surgery, Pancreas Institute, University of Verona Hospital Trust, Verona, Italy; Department of Surgery, Poliambulanza Hospital Brescia, Brescia, Italy; Department of Surgery, Regional Academic Cancer Centre Utrecht, University Medical Centre Utrecht, and St Antonius Ziekenhuis Nieuwegein, Utrecht, the Netherlands; Department of General and Pancreatic Surgery, Pancreas Institute, University of Verona Hospital Trust, Verona, Italy; Department of Surgery, Erasmus MC, Rotterdam, the Netherlands; Department of Surgery, Amsterdam UMC, University of Amsterdam, Cancer Centre Amsterdam, Amsterdam, the Netherlands

## Introduction

The incidence of postoperative pancreatic fistula (POPF) and morbidity after distal pancreatectomy (DP) is high^1–6^. Routine abdominal drainage aims to protect against severe consequences of POPF^7^, but the evidence for routine abdominal drainage after DP is unclear.

Most studies of drain management combined DP with pancreatoduodenectomy, and are therefore less useful^8–12^. Drain placement may lead to retrograde infection, patient discomfort, or direct damage to blood vessels^13^. A recent multicentre randomized trial^6^ demonstrated comparable outcomes with and without routine abdominal drainage after DP. It is unclear, however, whether omitting routine drainage in subgroups with a high risk of POPF would potentially lead to an increased risk of complications.

A systematic review of abdominal drainage after DP specifically is lacking. In this systematic review, the benefits and risks associated with a no-drain strategy *versus* abdominal drainage after DP were compared.

## Methods

A systematic review and meta-analysis was undertaken to compare no drain placement versus routine abdominal drainage in patients undergoing DP. The primary outcome was major morbidity, defined as complications with a Clavien–Dindo grade of III or higher. Secondary outcomes were POPF (International Study Group of Pancreatic Surgery (ISGPS) grade B/C, 2016)^1^, delayed gastric emptying (ISGPS grade B/C), postpancreatectomy haemorrhage (ISGPS grade B/C), radiological intervention, readmission, ICU admission, reoperation, and 30-day mortality. Meta-analysis was performed using Review Manager (RevMan) version 5.0 (The Cochrane Collaboration, Hamilton, Canada); details are available in *Appendix S1*.

## Results

The search identified 2176 studies, of which five^6,14–17^ were included involving 2153 patients, all of whom were included in the meta-analysis. The detailed search process is shown in *Appendix S2* and *Fig. S1*. *Tables S1* and *S2* show study characteristics, baseline characteristics, operative parameters, and outcome measures in each included study. Definitions in each study are detailed in *Table S3*. None of the included studies incorporated a subgroup analysis based on low or high POPF risk. Differences in (pre)operative and postoperative management are summarized in *Table S4*. Risk-of-bias assessment can be found in *Appendix S3*, *Fig. S2*, and *Tables S3–S7*.

### Meta-analysis

**Fig. 1:**
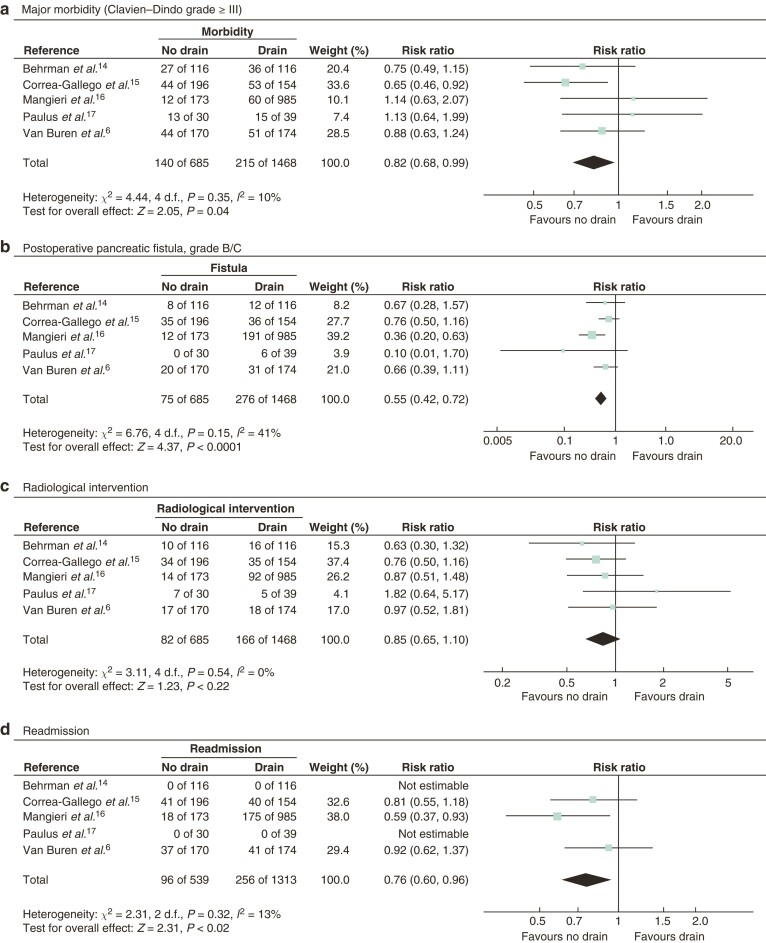
Meta-analysis of impact of no drain *versus* drain on outcomes after distal pancreatectomy **a** Major morbidity, **b** grade B/C postoperative pancreatic fistula, **c** radiological intervention, and **d** readmission. A Mantel–Haenszel fixed-effect model was used for meta-analysis. Risk ratios are shown with 95 per cent confidence intervals.

All five studies^6,14–17^ included data on the primary outcome, major morbidity, which was found to be lower in the no-drain compared with the drain group (risk ratio (RR) 0.82, 95 per cent c.i. 0.68 to 0.99) (*Table 1* and *Fig. 1*). There was no heterogeneity in the primary outcome between the studies.

**Table 1:** Summary of findings for no drain *versus* drain

Outcome	No. of studies	No. of patients	Statistical model	Risk ratio	Quality (GRADE)
**Major morbidity**	5	2153	M-H, fixed effect	0.55 (0.42, 0.72)	Moderate ⊕⊕⊕⊝
**POPF grade B/C**	5	2153	M-H, fixed effect	0.82 (0.68, 0.99)	High ⊕⊕⊕⊕
**Radiological intervention**	5	2153	M-H, fixed effect	0.85 (0.65, 1.10)	Moderate ⊕⊕⊕⊝
**Reoperation**	5	2153	M-H, fixed effect	0.93 (0.57, 1.51)	Moderate ⊕⊕⊕⊝
**Readmission**	3	1852	M-H, fixed effect	0.76 (0.60, 0.96)	Moderate ⊕⊕⊕⊝
**Alive at 30 days**	5	2153	M-H, fixed effect	1.00 (1.00, 1.01)	Moderate ⊕⊕⊕⊝
**Postpancreatectomy haemorrhage**	2	1502	M-H, fixed effect	0.98 (0.45, 2.15)	Moderate ⊕⊕⊕⊝
**Surgical-site infection**	1	232	M-H, fixed effect	1.86 (0.77, 4.49)	Low ⊕⊕⊝⊝
**Intra-abdominal abscess**	2	413	M-H, fixed effect	0.93 (0.53, 1.61)	Moderate ⊕⊕⊕⊝

Values in parentheses are 95 per cent confidence intervals. GRADE, Grading of Recommendations Assessment, Development and Evaluation with possible scores: very low: ⊕⊝⊝⊝, low: ⊕⊕⊝⊝, moderate: ⊕⊕⊕⊝ and high: ⊕⊕⊕⊕. M-H, Mantel–Haenszel; POPF, postoperative pancreatic fistula.

All five studies^6,14–17^ reported data on POPF grade B/C. Pooled analysis showed that the POPF rate was lower in the no-drain group compared with the drain group (RR 0.55, 0.42 to 0.72). Readmissions were reported in three studies^1,16,17^, with a lower rate in the no-drain group (RR 0.76, 0.60 to 0.96).

Rates of radiological intervention, postpancreatectomy haemorrhage, delayed gastric emptying, intra-abdominal abscess, surgical-site infection, reoperation, and 30-day mortality were no different between groups. Detailed results of the meta-analysis are shown in *Appendix S4*, *Table 1*, and *Fig. 1*.

## Discussion

No drain placement after DP was associated with a lower rate of major complications (Clavien–Dindo grade at least III), POPF, and readmissions. Rates of radiological intervention and reoperation did not differ. No study has reported on high-risk subgroups.

A few studies concluded that omitting drains after DP was safe, potentially because most of them analysed a combination of pancreatoduodenectomy and DP. POPF after pancreatoduodenectomy is different as there is, by definition, an infection owing to underlying anastomotic dehiscence. This cannot be compared with the situation after DP^8–11^.

Five studies were included in the present meta-analysis, which has a high statistical power and effect size by including a large number of patients. In the study by Paulus and colleagues^17^ the no-drain group had a lower rate of POPF (0 *versus* 15 per cent), without differences in other complications. The discrepancy between POPF and other complications in the no-drain group can be explained by use of the older terminology for POPF, which has been updated since then. This why severe morbidity was chosen as primary endpoint in the present study. Mangieri *et al*.^16^ reported a higher rate of POPF grade B/C and readmissions in the drain group. Behrman and co-workers^14^ reported no difference between groups in severe morbidity and grade B/C POPF. Correa-Gallego and colleagues also did not find any disadvantages in the no-drain group^15^. The only included randomized multicentre trial, by Van Buren *et al*., did not find a difference in rate of POPF, but noted comparable rates of radiological intervention between the groups^5^. This trial did not stratify by subgroups such as high- and low-risk POPF. It therefore remains unclear whether the outcomes reported in the present meta-analysis also apply to high-risk subgroups. This meta-analysis has confirmed the findings of Van Buren that a routine drain policy does not protect the patient from additional radiological interventions.

Recently, the first distal fistula risk score was constructed, which includes duct size and pancreatic thickness (M. v. B. E. De Pastena, submitted for publication). This prediction model enables the clinician to determine the risk of POPF, so that appropriate measures can be taken, such as selective drainage in high-risk patients. Future pragmatic multicentre randomized trials including risk-stratified randomization are required before final conclusions can be drawn.

This study had several limitations. Non-randomized studies could have been exposed to selection bias, information bias, and follow-up bias because patients who did not receive drains may have had favourable characteristics leading to omission of drains. The definition of POPF differed between studies. Potential bias was minimized by analysing only POPF grade B/C according to the ISPGS^1^. There was heterogeneity between studies. In most studies, however, no clinically relevant differences were observed between preoperative, perioperative, and postoperative parameters in the two groups, such as stump closure methods. Different stump closure methods could lead to a difference in POPF rate^18^.

## Supplementary Material

znac042_Supplementary_Data
