# Supplementary material for: Routine abdominal drainage after distal pancreatectomy: meta-analysis
Source: Br J Surg. 2022 Mar 30;109(6):486–8. doi: 10.1093/bjs/znac042 (PMC10364730; doi:10.1093/bjs/znac042)
Supplement: znac042_Supplementary_Data [file znac042_supplementary_data.zip › Supplementary_material.docx]

| **FIGURE S1.** PRISMA flow chart |
| --- |


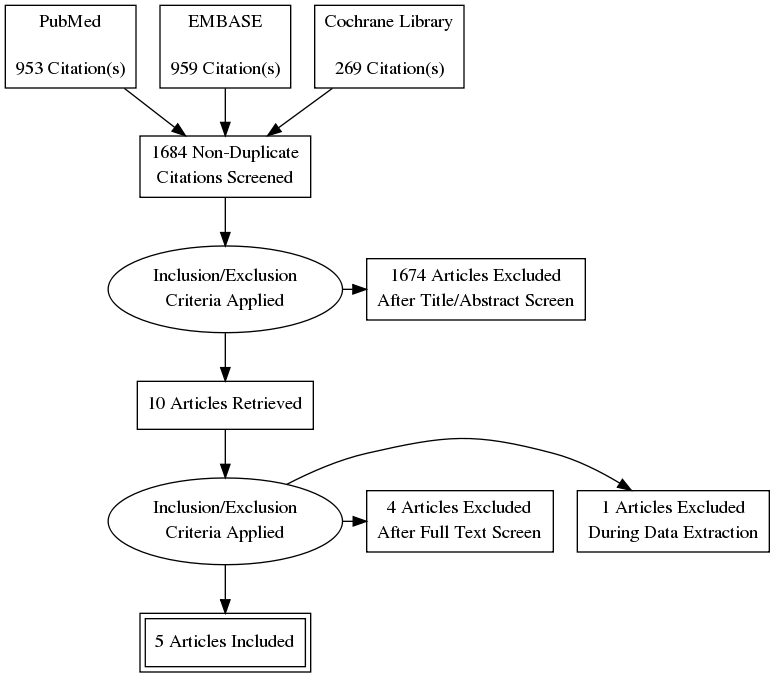


|  |
| --- |

| **FIGURE S2.** Funnel plot of primary outcome major morbidity |
| --- |

|  |
| --- |


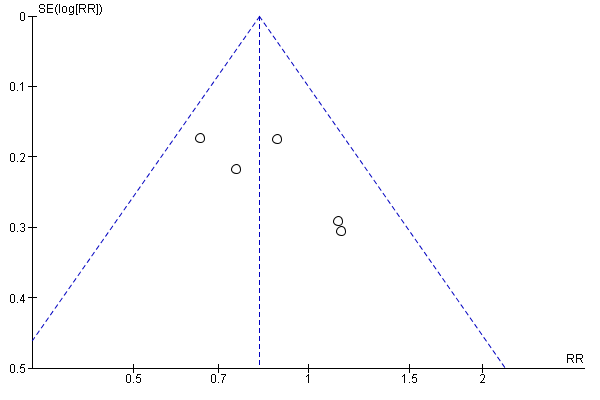


| **TABLE S1.** Study Characteristics | | | | | |
| --- | --- | --- | --- | --- | --- |
| **First author** | **Paulus** | **Correa** | **Behrman** | **Van Buren** | **Mangieri** |
| Year published | 2012 | 2013 | 2015 | 2017 | 2020 |
| Study design | Retrospective | Retrospective | Retrospective | Randomized trial | Retrospective |
| Country | USA | USA | USA | USA/Canada | USA |
| Design | Retrospective, monocenter | Retrospective, monocenter | Propensity score-matched, multicenter | Randomized controlled trial, multicenter | Retrospective, multicenter |
|  |  |  |  |  |  |
| Primary outcome | Postoperative morbidity and the need for therapeutic intervention | Morbidity, POPF | Serious morbidity | 60-day grade 2 or  higher grade complication rate | Major complications |
| Inclusion criteria | Elective distal pancreatectomy | Partial pancreatic resection (subanalysis for DP) | Elective distal pancreatectomy | Elective distal pancreatectomy | Distal subtotal pancreatectomy, with or without splenectomy |
| Exclusion criteria | Concurrent extrapancreatic organ removal exclusive  of the spleen | NR | NR | Age < 18 years | Preoperative jaundice, biliary stent or ASA grade 5; operations requiring pancreatic or enteric anastomosis, vascular resection, or biliary drain placement |
| Number of surgeons | 3 | 6 | NR | NR | NR |
| ASA: American Society of Anesthesiologists classification; POPF: postoperative pancreatic fistula; CV: Clavien-Dindo ≥ 3 complications; NR: not reported | | | | | |

| **TABLE S2.** Study Population Characteristics | | | | | | | | | | |
| --- | --- | --- | --- | --- | --- | --- | --- | --- | --- | --- |
| **First author** | **Paulus** | | **Correa** | | **Behrman** | | **Van Buren** | | **Mangieri** | |
|  | Drain | No drain | Drain | No drain | Drain | No drain | Drain | No drain | Drain | No drain |
| Patients | 39 | 30 | 154 | 196 | 116 | 116 | 174 | 170 | 985 | 173 |
| Male n (%) | 15 (38) | 15 (50) | 75 | 100 | 49 (42) | 55 (47) | 72 (41) | 67 (39) | 414 (42) | 65 (38) |
| Age mean, y | 52 | 58 | 65 | 65 | 57 | 59 | 61 | 61 | 62 | 58 |
| ASA 3 or higher n (%) |  |  |  |  | 75 (65) | 82 (71) | 129 (74) | 120 (71) | 654 (66) | 118 (68) |
| **Operative** |  |  |  |  |  |  |  |  |  |  |
| Operative time in min, median | 249 | 186 | 191 | 152 | 222 | 228 | 200 | 150 | 206 | 212 |
| eBL in ml, median | 450 | 195 | 400 | 200 |  |  | 196 | 203 |  |  |
| Laparoscopic n (%) |  |  |  |  | 32 (28) | 32 (28) | 77 (44) | 75 (44) | 531 (54) | 95 (55) |
| Conversion n (%) |  |  |  |  |  |  |  |  | 0 (0) | 0 (0) |
| **Postoperative** |  |  |  |  |  |  |  |  |  |  |
| POPF (%) | 6 (15) | 0 (0) | 36 (23) | 35 (18) | 12 (10) | 5 (4) | 31 (18) | 20 (12) | 191 (19) | 12 (7) |
| DGE (%) |  |  |  |  |  |  | 10 (6) | 9 (5) |  |  |
| PPH (%) |  |  |  |  |  |  | 2 (1) | 2 (1) | 35 (4) | 6 (3) |
| SSI (%) |  |  |  |  | 13 (11) | 7 (6) | 8 (5) | 5 (3) |  |  |
| IAA (%) | 8 (21) | 7 (23) |  |  |  |  | 16 (9) | 13 (8) |  |  |
| Radiological intervention (%) | 5 (13) | 7 (23) | 35 (23) | 34 (17) | 16 (14) | 11 (9) | 18 (10) | 17 (10) | 92 (9) | 14 (8) |
| Reoperation (%) | 11 (28) | 8 (27) | 1 (1) | 0 (0) | 1 (1) | 3 (3) | 9 (5) | 6 (4) | 27 (3) | 5 (3) |
| Readmission (%) |  |  | 40 (26) | 41 (21) |  |  | 41 (24) | 37 (22) | 175 (18) | 18 (10) |
| Clavien-Dindo ≥ 3 (%) | 15 (38) | 13 (43) | 53 (34) | 44 (22) | 36 (36) | 27 (23) | 51 (29) | 44 (26) | 60 (6) | 12 (7) |
| 30 day mortality (%) |  |  | 3 (2) | 1 (1) |  |  | 0 (0) | 0 | 5 (1) | 1 |
| LOS in days, median | 9 | 6.5 | 7 | 5 | 6 | 6 | 5 | 5 | 5 | 5 |
| ASA: American Society of Anesthesiologists classification; eBL = estimated blood loss in milliliter; POPF: postoperative pancreatic fistula; DGE: delayed gastric empyting; PPH: postpancreatectomy hemorrhage; SSI: surgical site infection; IAA: intra-abdominal abscess; LOS: length of hospital stay | | | | | | | | | | |

| **TABLE S3** Definitions Comparison Table | | | | | |
| --- | --- | --- | --- | --- | --- |
| **Study** | **POPF definition** | **Corresponding with** | **Serious morbidity definition** | **Corresponding with** |  |
| Paulus 2012 | ISGPS 2005. B/C included (A=0) | ISGPS B/C 2016 | Need for therapeutic intervention | Clavien-Dindo 3 or higher |  |
| Correa 2013 | Amylase-rich drain output + requiring IR, endoscopic or operative intervention. | ISGPS B/C 2016 | IR, endoscopic or operative intervention required | Clavien-Dindo 3 or higher |  |
| Behrman 2015 | ISGPS 2005 A/B/C requiring percutaneous drainage, reoperation or death. | ISGPS B/C 2016 | Corresponding with Clavien-Dindo 3 or higher | Clavien-Dindo 3 or higher |  |
| Van Buren 2017 | ISGPS 2005 B/C | ISGPS B/ C 2016 | CTCAE 3 or higher | Clavien-Dindo 3 or higher |  |
| Mangieri 2020 | ISGPS 2016 B/C | ISGPS B/ C 2016 | Clavien-Dindo 3 or higher |  |  |
| ISGPS: International Study Group Pancreas Surgery; CTCAE: Common Terminology Criteria for Adverse Events | | | | | |

| **TABLE S4** pre- and postoperative management | | | | | | | | | |
| --- | --- | --- | --- | --- | --- | --- | --- | --- | --- |
|  | Stump closing | Additional stump methods | Octeotride | Indication for drain | Matching variables | Location drain | Suction / what pressure | Protocol drain removal | Time to drain removal |
| Paulus | Electrocautery transection, ligation of the Wirsung duct, monofilament suture.  No stapling. | Omentum or the falciform ligament and  fibrin glue over the suture line. | All: 200 µg /8 h until discharge or for 1 week | No specific indication given. All drainless patients derived from 1 of 3 surgeons | NA | NS | Closed suction drainage. Pressure not reported. | Output less than 50 mL/d  and/or amylase < 3x normal serum level | NM |
| Correa | Stapled (72%)  Stump oversewn with duct ligation (28%) | Seamguard reinforcement in 34% and oversewn stump in 15% of stapled cases. | NM | 6 surgeons differed in their draining strategy. Rationale behind their choosing was not specified. | NA | NS | NS | NM | NM |
| Behrman + matching variabelen | NM | NM | NM | NM | age, gender, race, BMI, preoperative  albumin, pancreatic texture and duct size, organ or vascular resection, final pathology. | NS | NS | NM | NM |
| Van Buren | Stapling in 123 (71) vs 122 (72) Oversewn in 37 (21) vs 33 (19) # | Tissue link 0 (0) vs 6 (4) # | Not used | NA, RCT | NA | NS | Closed suction drainage. Pressure not reported. | Output less than 20 mL/d or  amylase < 3x normal serum level | NM |
| Mangieri | NM | NM | NM | NS | NA | NS | NS | NM | NM |
| NA: not applicable; NM: not mentioned; BMI: body mass index; RCT: randomized controlled trial; # compared drain group vs no-drain group | | | | | | | | | |

| **TABLE S5** Non-RCTs assessment using the Newcastle-Ottawa scale | | | | |  |
| --- | --- | --- | --- | --- | --- |
| **Criteria** | **Paulus 2012** | **Correa 2013** | **Behrman 2015** | **Mangieri 2020** | |
| A. Selection |  |  |  |  | |
| Exposed truly representative of average | 🟌 | 🟌 | 🟌 | 🟌 | |
| Selection of non-exposed from the same community | 🟌 | 🟌 | 🟌 | 🟌 | |
| Exposure ascertained by secure record or interview | 🟌 | 🟌 | 🟌 | 🟌 | |
| Demonstration of outcome of interest not present at the start of the study | 🟌 | 🟌 | 🟌 | 🟌 | |
|  |  |  |  |  | |
| B. Comparability |  |  |  |  | |
| Study controls for preoperative comparability | 🟌 | 🟌 | 🟌 | 🟌 | |
| Study controls for operative comparability |  |  | 🟌 | 🟌 | |
|  |  |  |  |  | |
| C. Outcome |  |  |  |  | |
| Adequate assessment and description of Outcome | 🟌 | 🟌 | 🟌 | 🟌 | |
| Was Follow-Up Long Enough for Outcomes to Occur | 🟌 | 🟌 | 🟌 | 🟌 | |
| Adequacy of Follow Up of Cohorts | 🟌 | 🟌 | 🟌 | 🟌 | |
| Score | 8 | 8 | 9 | 9 | |
|  | | | | |  |

| **TABLE S6** Randomized trial assessment using Cochrane Collaboration Handbook | |
| --- | --- |
| **Criteria** | **Van Buren 2017** |
| Random sequence generation (selection bias) | Low risk |
| Allocation concealment (selection bias) | Low risk |
| Blinding of participants and personnel (performance bias) | High risk |
| Blinding of outcome assessment (detection bias) | High risk |
| Incomplete outcome data (attrition bias) | Low risk |
| Selective reporting (reporting bias) | Low risk |
|  | |

| **TABLE S7** GRADE evidence table | | | | | |  |  |  |  |
| --- | --- | --- | --- | --- | --- | --- | --- | --- | --- |
| **Outcome** | **No of studies** | **Risk of bias** | **Inconsistency** | **Indirectness** | **Imprecision** | **Other considerations** | **Quality GRADE** | | |
| Major morbidity | 1 RCT 4 RS ^5, 6, 11-13^ | NS | p=0.15 I²=41% | NS | NS |  | High ⊕⊕⊕⊕ |  |  |
| POPF B / C | 1 RCT 4 RS ^5, 6, 11-13^ | NS | p=0.35 I²=10% | NS | NS | large magnitude upgrade | Mod ⊕⊕⊕⊝ |  |  |
| Radiological intervention | 1 RCT 4 RS ^5, 6, 11-13^ | NS | p= 0.45 I²=0% | NS | NS |  | Mod ⊕⊕⊕⊝ |  |  |
| Reoperation | 1 RCT 2 RS ^5, 11, 12^ | NS | p= 0.32 I²=13% | NS | NS |  | Mod ⊕⊕⊕⊝ |  |  |
| Readmission | 1 RCT 4 RS ^5, 6, 11-13^ | NS | p= 0.72 I²=0% | NS | NS |  | Mod ⊕⊕⊕⊝ |  |  |
| Alive at 30 days | 1 RCT 4 RS ^5, 6, 11-13^ | NS | p= 0.71 I²=0% | NS | NS |  | Mod ⊕⊕⊕⊝ |  |  |
| PPH | 1 RCT 1 RS ^5, 12^ | NS | p= 0.97 I²=0% | NS | NS |  | Mod ⊕⊕⊕⊝ |  |  |
| SSI | 1 RS ^6^ | NS | NA | NS | NS |  | Low ⊕⊕⊝⊝ |  |  |
| IAA | 1 RCT 1 RS ^5, 13^ | NS | p= 0.59 I²=0% | NS | NS |  | Mod ⊕⊕⊕⊝ |  |  |
| POPF: postoperative pancreatic fistula; DGE: delayed gastric empyting; PPH: postpancreatectomy hemorrhage; SSI: surgical site infection; IAA: intra-abdominal abcsess; RCT: Randomized controlled trial; RS: retrospective; NS: Not serious | | | | | | | | |  |

**Appendix S1: detailed methods**

A systematic review and meta-analysis was performed to compare no drain placement versus routine abdominal drainage in patients undergoing DP. An electronic search was performed by two independent reviewers (E.A.V.B. and T.V.R.) in PubMed, Cochrane Central Register of Controlled Trials, and Embase until January 1st, 2021. The search included 'distal pancreatectomy', 'drain', 'drainage' and synonyms and was restricted to the availability of English, Dutch, Italian, and German full-text. All identified publications were reviewed for inclusion by both reviewers (E.A.V.B. and T.V.R.) using predetermined eligibility criteria. Any inconsistencies were addressed by discussion and consensus among the two reviewers. References of the identified articles were crosschecked. The screening process was done according to the PRISMA statement.(16) A review protocol was developed and was registered with PROSPERO (number CRD42020222454).

*Eligibility criteria*

Inclusion criteria were randomized and non-randomized studies, prospective and retrospective studies comparing no drain versus routine abdominal drainage in distal pancreatectectomy; studies reporting on severe morbidity and POPF; studies in adults; and studies in the English, Dutch, Italian, and German language with availability of full-text. Exclusion criteria were: review articles, abstracts, editorials, case reports, and letters to the editor; incomplete data on outcomes of interest as Clavien-Dindo scores and POPF grades, defined by the ISGPS 2016 definition(3); and studies concerning other pancreatic surgery than DP.

*Outcomes*

The primary outcome of this study was major morbidity, defined as a Clavien-Dindo grade 3 complications or higher.(17) Secondary outcomes were POPF (ISGPS grade B/C 2016)(3), DGE (ISGPS grade B/C)(18), postpancreatectomy haemorrhage (ISGPS grade B/C)(19), radiological intervention, readmission, intensive care unit (ICU) admission, reoperation, and 30 day mortality.

*Data collection and analysis*

A meta-analysis was performed using Review Manager (RevMan software, version 5.0 (The Cochrane Collaboration)). Risk ratios were calculated with 95 per cent confidence intervals and a p-value of <0.05 was considered statistically significant. Heterogeneity was investigated with the chi-square and I2 test and interpreted as follows: 0 to 40 per cent low, 30 to 60 per cent moderate, 50 to 90 per cent high, and 75 to 100 per cent considerable. A fixed effects model was used with a I² index of lower than 50 per cent and a random effects model was used with I² > 50 per cent. A potential publication bias for the primary outcome was visually inspected by funnel plot.

*Data extraction and management*

A standardized data extraction form was used by the two independent reviewers. The following data were extracted from the included studies: First author, year of publication, study design, sample size of the groups, baseline characteristics, details of surgery, postoperative care, and outcomes.

*Risk of bias assessment*

Quality of the non-RCTs were assessed using the Newcastle-Ottawa scale(20) and quality of randomized trials were assessed with the Cochrane Collaboration Handbook.(21) The independent outcomes were assessed with the GRADE approach. Inconsistencies were assessed with the heterogeneity factor p and I2. Imprecision was calculated with the Optimal Information Size. Publication bias was assessed visually using a funnel plot.

Appendix S2: search results details

The search identified 2176 studies based on title and abstract and inclusion criteria. Overall, 2166 studies were excluded: 492 duplicates, 1512 studies wherein DP was not the subject of analysis, 162 studies because drain placement was not a variable. Of the remaining 10 studies, full text papers were obtained. Thereafter, one study was excluded because groups were not compared according to the presence of a surgical drain. Four more studies were excluded because no distinction of results was made between pancreatoduodenectomy and DP.^7-10^ No studies were added after a crosscheck. Finally, five studies remained. ^5, 13-16^ A flowchart of the literature search is shown in Figure 1.

### Appendix S3: Risk of bias

In two studies, the groups did not differ in preoperative characteristics but differences were seen in operative characteristics. In the other three studies, no differences were seen in both preoperative and operative characteristics between the groups. These differences between drain and no drain groups were included in the quality assessment shown in Additional Tables 5 and 6. The independent outcomes were reviewed in the GRADE evidence table (Additional Table 7) where they are displayed with their effect on the outcomes. By following the GRADE approach, the observational studies were collectively upgraded from low to moderate certainty because of the large magnitude of effect for POPF (RR: 0.46). For major morbidity and POPF, definitions per study were displayed in Additional Table 3 to assess if the used outcome definitions of the studies can be compared. For all five studies, the definitions used to report severe morbidity correspond with Clavien-Dindo 3 or higher. For all five studies the definitions used to report POPF correspond with the ISGPS grade B/C 2016 definition.^1^ Therefore, these definitions can be compared and analysed as equal.

Inconsistency was determined based on the heterogeneity factor p and I^2^. Most confidence intervals overlapped. For the outcomes POPF, major morbidity, and radiological intervention the inconsistency was found to be low. There was no heterogeneity between groups. The preoperative parameters / baseline characteristics could not be pooled for assessment of homogeneity. Therefore the homogeneity of the groups was assessed per study out of which could be concluded that the studies compared homogeneity groups. The directness of the studies was assessed using the PICO format and was found high. The included patients do well represent the overall patient population who will undergo DP. The outcomes are clinically relevant as Clavien-Dindo ≥ 3 gives an overall view of major morbidity, POPF is still the most important complication after DP and radiological intervention determines whether an operative drain is successful in the prevention of adverse events which require postoperative interventions (such as intra-abdominal abscess, POPF, postpancreatectomy haemorrhage). A potential weakness in the directness could be the surgical developments, for instance the increasing use of robot-assisted DP. To determine if imprecision was an influence on the quality of the studies, the Optimal Information Size was calculated using the GRADE approach for the outcome of major morbidity. With an event rate of 28 per cent Clavien-Dindo ≥ 3 complications, the optimal information size implicates a sample size of minimally 460 is required. The reasonable threshold is met and therefore no downgrading was performed as shown in Table 1. The risk of publication bias was assessed by a funnel plot for the primary outcome major morbidity as shown in additional Figure 2.

Appendix S4: details of meta-analysis

All five studies reported data on POPF grade B/C.^5, 13-16^ Two studies defined their POPF using the ISGPS 2005 definitions, one trial used the ISGPS 2016 definitions. The definitions per study are displayed in Additional Table 3. Since all trials reported only the clinically relevant fistula, the data could be pooled.^5, 13-16^ Pooled analysis showed that occurrence of POPF was significantly lower in the no drain group compared to the drain group (RR 0.55 [0.42, 0.72] p<0.001).

Radiological interventions was reported in all five studies and showed no significant difference between the no drain group and drain group (RR 0.85 [0.65, 1.10] p=0.22].^5, 13-16^

Readmission was reported in three studies. The readmission rate was significantly lower in the no drain group compared to the drain group (RR 0.76 [0.60, 0.96] p=0.02).^5, 14, 15^

Postpancreatectomy haemorrhage was reported in two studies and was not found to be significantly different between groups (RR 0.98 [0.45, 2.15] p=0.97).^5, 15^

Delayed gastric emptying was reported in one study and was not found to be significantly different between groups (OR 0.92 (0.36–2.32) p=0.85).^5^

Intra-abdominal abscess was reported in two studies and was not found to be significantly different between groups (RR 0.93 [0.53, 1.61] p=0.78).^5, 16^

Surgical site infections was reported in two studies and was not found to be significantly different between groups (RR 1.21 [0.62, 2.35] p=0.57).^5, 13^

Reoperations were reported in all five studies and showed no significant difference between both groups (RR 0.93 [0.57, 1.51] p=0.76). ^5, 13-16^

The 30-day mortality was reported in all five studies. ^5, 13-16^ However, in two studies there were no deaths in both groups so it could not be included in the meta-analysis. To include all studies which reported data on 30-day mortality, 30-day alive was analysed and was not found to be significantly different between groups (RR 1.00 [1.00, 1.01] p=0.37). ^5, 13-16^
